# Tree adaptive growth (TAG) model: a life-history theory-based analytical model for post-thinning forest stand dynamics

**DOI:** 10.3389/fpls.2024.1344883

**Published:** 2024-02-26

**Authors:** Bernard Roitberg, Chao Li, Robert Lalonde

**Affiliations:** ^1^ Department of BioScience, Simon Fraser University, Burnaby, BC, Canada; ^2^ Canadian Wood Fibre Centre, Canadian Forest Service, Edmonton, AB, Canada; ^3^ Department of Biology, University of British Columbia, Kelowna, BC, Canada

**Keywords:** compensatory growth, thinning, life history theory, model, forestry, overcompensation

## Abstract

**Background:**

Understanding stand dynamics is essential for predicting future wood supply and associated ecosystem services for sustainable forest management. The dynamics of natural stands can be characterized by age-dependent growth and yield models. However, dynamics in managed stands appear somewhat different from that of natural stands, especially with difficulties in explaining the phenomenon of post-thinning overcompensation, based upon some long-term observations. Though overcompensation is an ideal outcome for the forest sector, it had been largely treated as an outlier and thus ignored or dismissed as “out-of-the-ordinary”.

**Methodology:**

We developed a life history theory-based, state-dependent model of Tree Adaptive Growth (TAG) to investigate this phenomenon and verified that overcompensation should be a common outcome in post-thinning forest stands when the stand growth over time is sigmoid shaped. TAG posits that individual trees will invest proportionately more into growth following thinning because it is evolutionarily adaptive to do so.

**Results:**

Our investigation of the model’s behavior unearthed diverse stand growth patterns similar to that which is observed in the empirical datasets and predicted by a statistics-based Tree’s Compensatory Growth (TreeCG) model.

**Conclusion:**

A simple, theory-driven, analytical model, TAG, can reproduce the diverse growth patterns in post-thinning stands and thus assist addressing silviculture-related issues. The model can be applied to various jurisdictions even without detailed regional growth and yield relationships and is capable of incorporating the effects of other time sensitive factors like fertilization, pruning, and climate change.

## 1 Introduction

Forests are extensively managed for the harvesting of wood, a material which provides many of the necessities of daily human life. In addition to this, forests are valued for their ecosystem services, which protect and maintain healthy environments. The dynamics of forests are a product of interactions between the growth of trees or forest stands and their environment, which includes physical site variables, natural disturbances such as fire and insect attack, as well as anthropogenic disturbances, which includes harvesting practices (Davis et al., 2001). Disturbances may result in immediate negative impacts on forests, which would compromise many of the benefits that forests provide. Our study focuses on the effect of thinning, a management practice that is a common anthropogenic disturbance, on forest stand dynamics.

Regional economic development often drives an increase in demand for wood materials and wood products (Buongiorno et al., 2003), and therefore presents a significant challenge to forest industries to maintain sufficient wood supply. To prevent over utilization of forest resources, a major component of forest management is to regulate harvest activities through annual allowable cut (AAC) for maintaining forest resources in a sustainable manner (Leuschner, 1984). An essential requirement for this AAC determination is a good understanding of long-term stand growth dynamics. Consequently, various growth functions have been estimated for natural stands of different tree species, site conditions, and geographical regions. However, the resulting growth curves necessarily are site-, and region-specific and lack generality, making prediction difficult as data acquisition on the local scale is logistically challenging. As a compromise, an assumption of same stand dynamics in natural and managed stands is generally made when developing management tools such as Woodstock of Remsoft (https://remsoft.com/woodstock-optimization-studio/) and Patchworks of Spatial Planning System (https://spatial.ca/patchworks/). Consequently, there is a need to improve our general understanding of stand dynamics in order to make accurate AAC estimation for managed stands in the context of anthropogenic disturbance.

A focus on disturbance tools for enhancing forest productivity will not only increase raw wood materials but also improve associated ecosystem services. This approach essentially addresses the question of “is it possible to increase natural growth of forest stands by judicious removal of some trees?” (Zeide 2011) that has attracted foresters for centuries. In forestry practice, a significant challenge for forest landowners and managers is the low productivity of forests which is a consequence of the slow growth rate of trees. Consequently, forest managers seek ways to speed up the growth and renewal of forest resources; tree removal or thinning is one such approach.

Disturbance ecology has elucidated how disturbances can affect tree and stand growth. However, the mechanistic responses of individual trees or stands of trees to disturbances remains poorly understood (McKenney, 2000). This presents significant challenges to sustainable forest management (SFM) decisions and carbon and GHG emission estimation because of the uncertainty involved in the post-thinning stand dynamics represented by the growth and yield relationships in managed stands. As an anthropogenic disturbance, thinning is a widely applied silviculture treatment for stand density management to produce the larger diameter trees that are desired by lumber industries. On one hand, thinning causes immediate loss of standing volume; and on the other hand, thinning promotes the accelerated growth of remaining trees (e.g., Bose et al., 2018).

There is a large literature focusing on thinning, in particular pre-commercial thinning (PCT) of smallest trees, the focus of this paper, which is mostly based on short-term observations as often required by guidelines (e.g., Reukema, 1975; Sohn et al., 2014; Elfstrom and Powers, 2023). A general conclusion Zeide (2011) reached as “after centuries of research and observations, we have learned that thinning, mostly from below, can increase merchantable but not total volume increment of trees per unit area”, and he continued “this knowledge is valuable but not satisfactory”. This summary reinforced Johnstone (1985) conclusion that “in all of the trials, the basal area and total volume of the thinned plots is well below that of the unthinned plots, but sufficient time has yet to elapse since treatment to indicate whether the thinned plots will ever catch up to their unthinned counterparts”.

By contrast with outcomes from these short-term observations, investigation into long-term silviculture datasets demonstrated that gross volume in thinned stands could exceed that in unthinned stands. For example, Steele (1955) found that the volumes from a thinned stand exceeded that from the control stand for a 20-year dataset of two young Douglas-fir stands in Skamania County of Washington, United States. This was also demonstrated in balsam fir (*Abies balsamea* (L.) Mill.) stands 42 years after thinning operations in the Green River of New Brunswick, Canada (Pitt and Lanteigne, 2008), and coastal Douglas-fir (*Pseudotsuga menziesii* [Mirb.] Franco) stands 40 years after initial treatments in the Shawnigan Lake of British Columbia, Canada (Li et al., 2018). In general, not enough attention has been paid to such results due to the difficulties in incorporation with existing results. Although these findings are derived from single sites, they nonetheless support Zeide’s (2011) suggestion that proper thinning can lead to enhanced stand productivity. To answer this question, a better understanding of the underlying mechanism(s) that generate this phenomenon is needed. This is where compensatory growth comes into play.

Compensatory growth (CG), a common cross-taxa phenomenon observed in both animals and plants, is defined as the accelerated growth of organisms after experiencing a period of unfavorable conditions (Mangel and Munch, 2005). CG has been well studied in crop and animal husbandry and is used to enhance productivity (Li et al., 2021), but it is a relatively new concept for forest practitioners. In forests, CG can be defined as accelerated forest stand growth after a stand experiences a loss of a proportion of the individual trees in a stand (Li et al., 2020). Li et al. (2018) used this concept to explain the enhanced costal Douglas-fir stand productivity in pre-commercial thinned stands 40-years after initial treatments. Recently, we described how CG following PCT has the potential to enhance forest yields (Li et al., 2020, 2021). We followed those synthetic reviews with data analysis that confirms that CG can predictably enhance productivity in real world forests (Li et al., 2022a). A simulation model named TreeCG, standing for Tree’s Compensatory Growth, was developed (Li et al., 2022b) to show how existing statistical growth and yield relationship from natural stands can be used to predict diverse stand growth curves in managed stands. As such, the observation of overcompensation, defined as the biomass in treated sites exceeds that in untreated sites, should not be seen as “out-of-the-ordinary”, but an understandable and predictable phenomenon.

The development of the TreeCG model was based on the variable-density yield table of lodgepole pine (*Pinus contorta* Douglas) (Johnstone, 1976) and can, in theory, be calibrated to different tree species and geographical regions using detailed local growth and yield relationships. However, this calibration is limited by the fact that not every jurisdiction has such detailed information readily available. Therefore, a logical next step is to develop a simple analytical CG model to eliminate the need for extensive calibrated datasets in order to apply this method to a wide variety of tree species and habitats. The key feature that allows one to move beyond the data is the incorporation of life history theory (LHT).

Here, LHT is used to build and implement a PCT – CG model that follows growth of individual trees in stands. LHT attempts to elucidate how natural selection designs organisms to maximize reproductive success, given knowledge of how selective factors in the environment (i.e., extrinsic mortality) and factors intrinsic to the organism (i.e., trade-offs, constraints) affect survival and reproduction (e.g., Stearns, 2000; Del Giudice et al., 2015; Simon et al., 2016), from an evolutionary biology perspective. In other words, it predicts how individual trees should invest in growth and reproduction under different circumstances, for example following a disturbance, to maximize their evolutionary fitness. It has successfully explained phenomena such as mast fruiting in trees (Lalonde and Roitberg, 1992) that were difficult to interpret with other theories. Elucidating the LHT response of individual trees to their environment allows one to extrapolate to the growth dynamics at the stand level.

Here, we present such an LHT-based state-dependent forest growth model. It assumes that trees will vary their energy allocations to growth, reserves, and reproduction as a function of physiological state (age, size and current reserves) and ecological circumstances (e.g., crowded versus thinned sites) in an adaptive manner i.e., to maximize lifetime reproductive success. This paper provides a proof of concept, state-dependent LHT model that can generate stand dynamics that mimic those observed in nature. Once confirmed, we then use this type of model to examine how optimal intensity and timing of PCT can enhance forest productivity.

The objectives of this manuscript are: (1) to present the LHT-based state-dependent forest growth model to show that this simple model can generate dynamics that are like those observed in nature; (2) describe the model behavior through a series of sensitivity analyses, to show how different factors could influence the outcomes of post-thinning stand dynamics; and (3) discuss the trade-offs of different approaches of modeling stand dynamics and their management applications.

## 2 Materials and methods

### 2.1 Theory rationale

As discussed in the Introduction, our model of thinning-driven stand dynamics is based upon state dependent life history theory for individual trees, which are then extrapolated to the stand level. We focus on three trees states: size (S), reserves (R) and age (A) whose units are defined as kg, kg and years, respectively (in section 2.2, Equation 12, we transcribe size to volume to bring in line with traditional forestry approaches). A fourth state variable, competitive ability (ψ), enters into stand-level dynamics described in the following paragraph. How trees respond to A, S and R states is modelled via incorporation of four important life history assumptions: (i) innate tree growth follows a sigmoidal pattern (see Weiner and Thomas, 2001), (ii) based upon principles from evolutionary ecology, we assume that tree growth strategies have evolved to maximize lifetime reproductive success (Davies and Krebs, 1993), (iii) individual trees will acquire resources and modify their investment in growth in a state dependent manner according to (S), (R), (A) (see Equations 1-3 below) and (iv) there are no genetic constraints on the above i.e., we play the phenotypic gambit (Grafen, 1984), which assumes that selection on phenotypic variation translates directly into selection on heritable variation in the population. Finally, though we do not explicitly say so, we assume that there is an underlying tradeoff between growth and reproduction (e.g., Kassaby and Barclay, 1992) though we acknowledge that such a tradeoff is often not obvious (e.g., Harshman and Zera, 2007; Suzuki et al., 2019).

We assume that individual trees at a given combination of our three state values (S, R, A) will adopt a resource allocation strategy. Thus, a focal tree, n, in population of N trees with state variables S = s, R = r and A = a will grow in an adaptive manner as follows:

We expect that trees will mobilize reserves for growth and reproduction, at a rate that we define as alpha (α).


(1) $$\alpha =\frac{r-R_{\min}}{1+e^{-\left(w_{1}s+w_{2}a\right)}}$$

Where: R_min_ is the minimum reserves value for a tree of size (s), with s, r and a defined as above and W values are weight constants for the state variables (see **Table 1** for a list of variables and their values). For a tree of a given size (s), we expect that α will increase with size, reserves and age up to some maximum that never causes the focal tree to exceed its critical reserves state value (R_crit_), which is the minimal level to maintain metabolic function. Here our reasoning is: (i) trees cannot forecast weather conditions for the current year and (ii) trees are risk averse and will never mobilize so many reserves so to put their survival at risk should the current year’s weather turn out to be poor for energy harvesting (see Clark’s asset protection principle, Clark, 1994).

**Table 1:** List of parameters employed in life history functions and TAG simulation model.

Parameters and Functions	Interpretation	Range
S	Size (kg)	15, 2800
R	Reserves (kg)	0.75, 560
A	Time since monitoring (year)	0, 199
α	Reserves mobilization function	Equation 1
w_1_	Size weight constant (α)	0.05
w_2_	Age weight constant (α)	0.05
β	Reproduction vs growth function	Equation 2
w_3_	Size weight constant (β)	0.007
w_4_	Age weight constant (β)	0.015
φ	Growth vs reserves function	Equation 3
H	Risk aversion	1.2
γ	Growth increment function	Equation 4
ξ	Mobilization efficiency	0.8
Λ	Cone production function	2.000 seeds/kg
ς_s_	Weather intensity vector	0.83, 1.2
ς_s_	Weather frequency vector	0.05, 0.4
ρ	Intrinsic growth rate	1.12
θ	Tree growth rate shape parameter	0.6, 1.4
ζ	Tree senescence	0.004
τ’	Density dependence shape parameter	0.2, 0.4
N	Stand population size	0, 100/0.04 ha
ψ	Relative competitive ability function	Equation 6
Κ	Growth from reserves function	Equation 7
δ	Total maintenance costs function	Equation 8
ω	Maintenance costs per mass unit	0
π	Cost to initiating reproduction function	Equation 9
ϵ	Reproduction cost slope	0.05
ϵ’	Reproduction cost intercept	0.1
V	Volume	0, 6.15 m^3^
D	Tree density	455 kg/m3

We define beta (β) as the proportion of mobilized reserves that go to reproduction versus growth:


(2) $$\beta \left(s,r,\alpha \right)=\{\begin{array}{cc} 1-e^{-\left(w_{3}s+w_{4}a\right)} & ifr>\pi \\ 0 & \text{otherwise} \end{array}$$

Where π is the start-up cost for initiating reproduction and the W’s are weight constants for size and age states (**Table 1**).

Here, we assume two effects of size. First, it is necessarily true that small trees have small reserves such that R will rarely exceed π, meeting the β = 0 condition. Second, small trees are not efficient at producing seeds (e.g., Sherman et al., 2019) thus, we have set the size weight (W_3_) low (**Table 1**). Regarding age weight (W_4_), we further assume that older trees should strongly bias their mobilized reserves to reproduction because future discounting (e.g., Lee et al., 2011) will offset small gains from growth particularly if older trees are large, thus, we employed a small value for W_4_.

We define phi (φ) as the allocation of harvested energy to growth versus reserves.


(3) $$\phi =\{\begin{array}{ll} \left(\frac{1}{\phi_{s}+\phi_{r}}\right)\phi_{s} & \;\text{if }r'<s'R_{max}\text{ and }r'<s'R_{min}\\ 1-\frac{r-\alpha +\gamma -sR_{max}}{\gamma } & \;\text{if }r'>s'R_{max}\\ 1-\frac{\left(s+\gamma \right)R_{min}-r^{\prime }}{\gamma } & \;\text{if }r'<s'R_{min} \end{array}$$

where: $\phi_{s}=1-\frac{s-s_{min}}{s}$ (equation 3.1) and $\phi_{r}=1-\left(\left(r-\left(R_{min}\right)\right)r\right)$ (equation 3.2), based upon size and reserves, respectively. In addition, $s^{'}=s+\gamma \left(\frac{1}{\phi_{s}+\phi_{r}}\right)\phi_{s}+\left(\alpha \left(1-\beta \right)\xi \right)$ (equation 3.3) and $r^{'}=r+\gamma \left(\frac{1}{\phi_{s}+\phi_{r}}\right)\phi_{s}-\alpha$ (equation 3.4) are potential updated values for size and reserves, respectively and ξ is defined as the efficiency in mobilizing reserves to structure (see Equation 7).

Here, three conditions hold: Sufficient reserves remain after metabolic costs and reproduction start-up cost are paid and allocation to reserves does not exceed the maximum reserve level after adjusting for energy harvesting (γ). Otherwise, φ is adjusted to meet those minimum and maximum reserve levels, respectively with γ and π being functions of tree size. The rationale for the φ decision is: as trees increase in size, their growth rate decelerates (Weiner and Thomas, 2001) thus there is little gain from investing in growth vs building up reserves for reproduction (Lalonde and Roitberg, 1992).

All three of the above state-dependent strategies can be summarized as: invest in growth when that leads to accelerated returns but only when reserves are sufficiently high to avoid starvation i.e., metabolic reserves fall below critical level. When trees are large and growth is constrained, trees are assumed to invest in reproduction when it is safe to do so because of the greater return on investment for the latter. Also, future discounting means that future reproductive returns from current investment in growth will be constrained for older and larger trees (Equation 2).

We employed Equations 1-3 in simulations of stage-dependent tree growth, which we refer to as Tree Adaptive Growth or TAG. Details of the TAG model follow.

### 2.2 TAG model development

**Figure 1:**
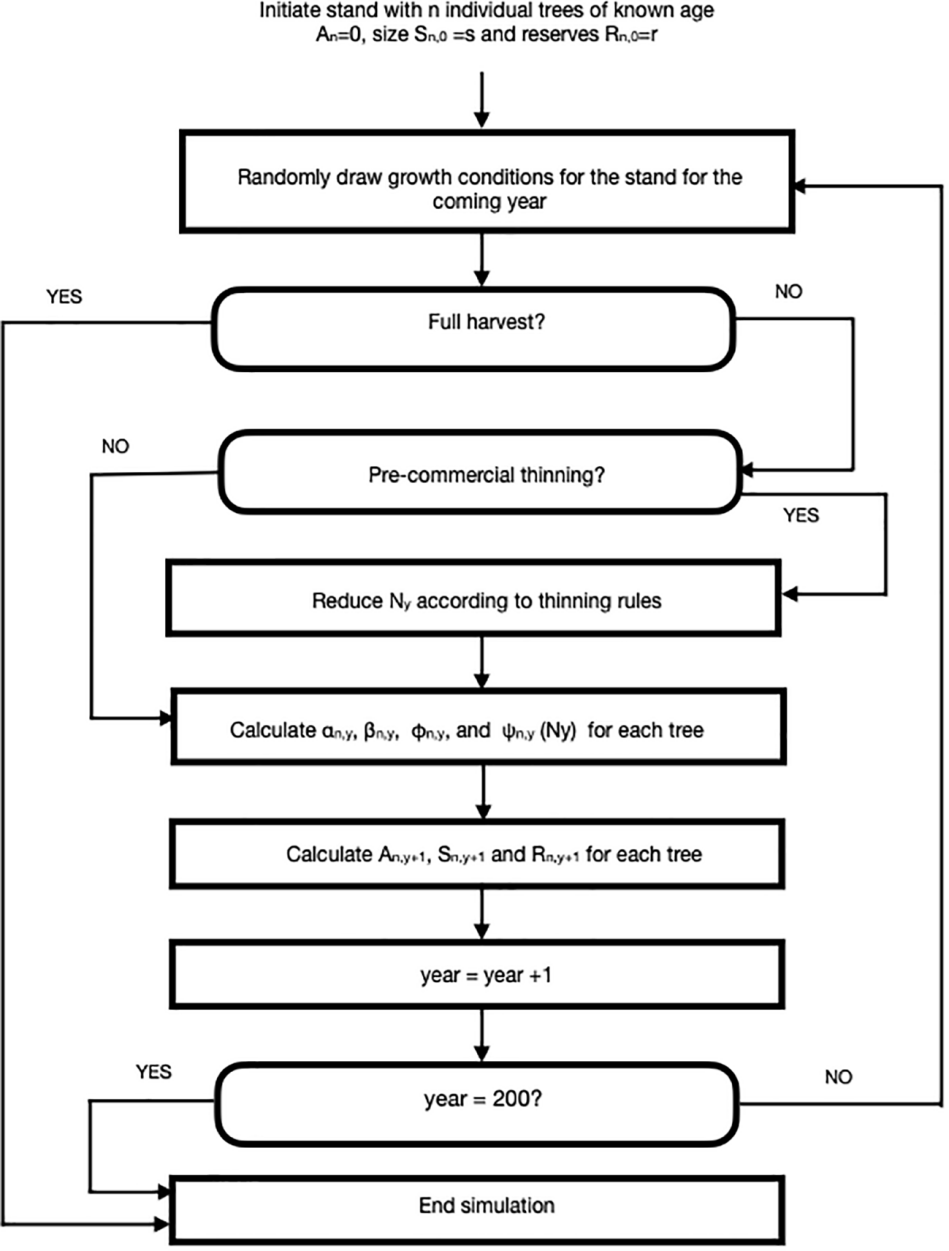
A flow chart for an agent-based Tree Adaptive Growth (TAG) simulation model. Description of parameters and their default values can be found in **Table 1**.

We developed an agent-based simulation model using C++ language, which is shown in flow chart form in **Figure 1**. Here, we simulated small stands of (N = 100 giving a starting density of 2500 stems/ha) trees that were subjected to silvicultural practices including pre-commercial and commercial thinning. We assumed that each stand was comprised of lodgepole pine (*Pinus contorta*) though we expect that our model could be employed for any tree species with known growth parameters or, potentially, mixed stands. The TAG model is similar in spirit (but not detail) to the DESPOT model of Buckley and Roberts (2006) in that individual trees are assumed to maximize some goal, in their case carbon gain and in ours, lifetime reproductive success.

Each stand was planted with identically aged individuals at year=0. The size (mass in kg) of each tree was randomly generated from a normal distribution (x=15, sd=5). Tree reserves (mass in kg) were generated from a uniform distribution (1,5). The simulation tracks size (S), reserves (R) and cone production (Λ), if any, each year for 200 years.

We employed a ‘weather’ concept to describe conditions that impact growth i.e., energy and nutrients that are harvested by trees for growth and maintenance. Our weather concept was created for characterizing the distribution/spectrum of favorable to unfavorable environmental conditions and such causative agents as variation in hydrological factors, heat stress or insolation. As many (most) of these factors show strong autocorrelation, we chose to use a single parameter in this proof-of-concept model, rather than breaking ‘weather’ into component effects.

At the beginning of each year, weather is randomly drawn from two vectors ς_s_ and ς_f_, which refer to weather intensity and frequency, respectively (see **Table 1** for details). Tree growth for any individual tree is a product of growth potential (Equation 4) and weather intensity for that year. We assumed that, in a given year, weather intensity is identical for every tree in a stand i.e., our model is aspatial (see Discussion).

At the beginning of each year, each tree is subjected to a random mortality event by individually drawing from a uniform distribution (0,1). If the random number falls above a preset default- annual-survival value, then the tree dies. For our runs, the default value for annual survival was set at 0.998. While this survival value is a constant, it is also possible for a tree to die if its reserves fall below a critical value, R_crit_, which would happen most frequently when an individual’s R state is low, weather is poor (see **Table 1**) and the focal individual is a poor competitor (Equations 5 and 6), thus the assumption of risk averse investment and reproduction as above. In these simulations, we assumed that R_min_ = R_crit_ H.

where: H describes the degree of risk aversion.

Following the mortality evaluation, the time counter for the current year was compared with the pre-determined time step for PCT (e.g., year = 15). If the current year was determined to be a PCT year, then individual trees were removed according to specific rules - in default simulations, the quartile of smallest trees were removed i.e., thinning from below; other thinning rules were also applied as discussed in section 2.3.vii. Late PCT was applied in a similar manner for trees later in the simulation.

Trees that survived random mortality and were not thinned were allowed to grow according to a modified logistic model where the potential growth increment for a tree of size (s) was:


(4) $$\gamma =\left(\rho -1\right)\left(1-{\left(\frac{s}{S_{max}}\right)}^{\theta }\right)\left(e^{-\varsigma y}\right)$$

Where: ρ is the intrinsic growth rate, S_max_ is the maximum mass that an individual tree can realize (but see Bontemps and Duplat, 2012; Stephenson et al., 2014), θ is a shape parameter and ς is a senescence term that describes decreasing energy harvesting with age (e.g., Hubbard et al., 1999; Qiu et al., 2021 but see Lanner and Connor, 2001).

**Figure 2:**
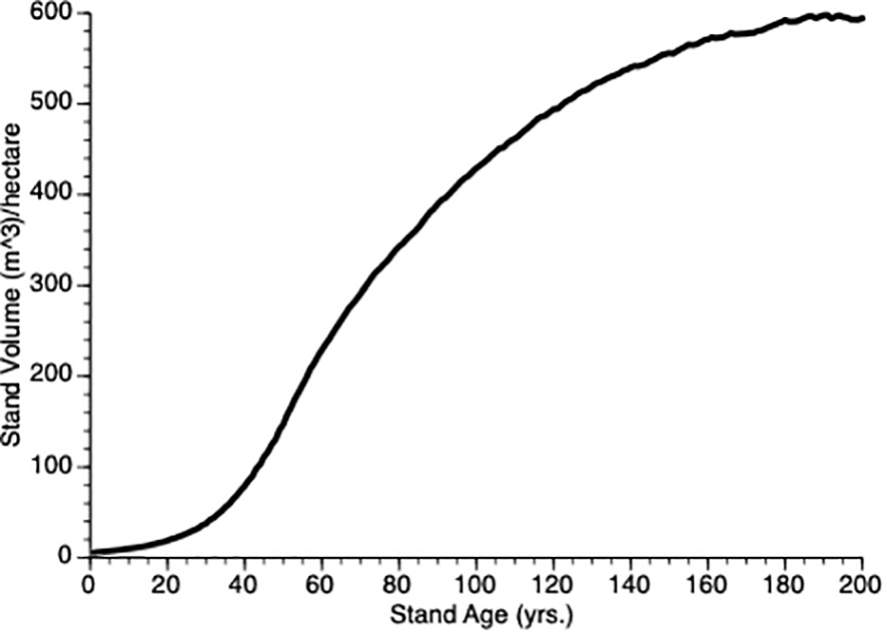
Mean stand volume over time from 5 TAG simulated unthinned (control) stands.

To determine our default value for ρ, we initiated simulations with ρ= 1.0 and then iterated a series (1.0, 1.20), increasing by 0.01 for each iteration and then plotting the stand volume against time from 0 to year 200. Since our goal was to produce a representative growth curve we visually confirmed that the TAG stand dynamics produced a sigmoidal plot as shown by existing growth and yield models like GYPSY (Huang et al., 2001, 2009) in Alberta (AB) and VDYP7 (British Columbia Ministry of Forests, Lands, Natural Resource Operations and Rural Development, 2019) in BC. Three features that we considered were, (i) the point of obvious acceleration in the growth curve, (ii) the inflection points (representing the change from the accelerating to the decelerating part of the growth curve) and (iii) maximum value of the growth curve for an unthinned (control) plot. In our case, the 15 m^3^/ha, year 50 and 600 m^3^/ha were used, respectively. **Figure 2** shows the TAG output that that we used based upon these criteria. In the end, we chose ρ = 1.12, the value that gave good representation of stand dynamics. In the default model, we assumed that θ =1 i.e., there is a linear decline in per unit mass growth with tree size, yielding a classic symmetric growth increment curve. In some simulations, we modified θ to give right or left skew to the size performance curve.

We further modified an individual’s potential growth increment from energy harvesting by including competitive performance. We assumed maximum performance for a tree with no competitors whose value declines with increasing density of competitors over time according to the function:


(5) $$\tau =1-(\frac{N_{y}}{N_{0}}{)}^{\tau \prime }\psi$$

Where: N_0_ is the number of trees at planting (year = 0) and N_y_ is the number of trees at year y and τ’ is a shape parameter for the function and ψ_s,n,y_ is relative competitive ability of a tree, n, of size s in year y, which is defined as:


(6) $$\psi_{s,n,y}=\frac{s_{n,y}}{{\bar{s}}_{y}}$$

Where: s_n,y_ is the size of a focal individual tree n at year y and S_y_ is mean tree size for a stand at year y.

Once the potential growth increment was calculated for a given tree of a given size (s) and reserve (r), new size and new reserve state values were calculated according to the three allocation rules (α, β and φ) as discussed in section 2.1.

A tree can also increase its structural mass by mobilizing reserves (β< 1 Equation 2). In doing so, we assume that there is some inefficiency in such mobilization, which we define as ξ i.e., ξ< 1.0. Thus, growth from reserves in year y is:


(7) $$\kappa ={\text{R}}_{\text{y}}\beta_{\text{y}}\xi_{\text{y}}\alpha_{\text{y}}$$

Reserves are decremented when trees pay maintenance costs, which we assume are size dependent. Thus, for a tree of size S, maintenance costs are:


(8) $$\delta_{s}=n_{S}\omega$$

Where: ω is the per unit mass cost of maintenance I n this version of our theory, we set ω to 0 based upon the assumption that maintenance costs are already subsumed in the growth function (Equation 4). Future work, may explicitly separate the two parameters.

In years in which an individual invests in reproduction, i.e., β > 0, we assume that there is a linear size dependent cost to reserves from initiating reproduction, which we define for a tree n of size S in year y as:


(9) $$\pi =\epsilon n_{s}+\epsilon^{\prime }$$

Where: ϵ and ϵ’ are the initiation slope and intercept, respectively.

From Equation 1 through 9, we can calculate (Equation 10) the size and reserves (Equation 11), respectively of an individual tree n in year y+1 based upon its size in year y as:


(10) $$s_{n,y+1}=s_{n,y}+\left(\gamma \tau \varsigma \phi \right)+\kappa$$

and


(11) $$r_{n,y+1}=\{\begin{array}{cc} r_{n,y}+\left(\gamma \tau \zeta \left(1-\phi \right)\right)-\left(r_{n}\gamma \alpha \right)-\delta_{n,sy}-\pi & \text{if}\beta >0\\ r_{n,y}+\left(\gamma \tau \zeta \left(1-\phi \right)\right)-\left(r_{n}\gamma \alpha \right)-\delta_{n,sy} & \text{otherwise} \end{array}$$

The simulation produces an even-aged stand of trees whose size distribution changes from year to year. There are several factors that generate individual size differences among trees. First, trees vary in size and reserves at initiation. From there, based upon absolute and relative size, and reserves, trees vary in their capacity to harvest energy but also in their tendency to allocate their harvest to growth, reserves and reproduction.

The process was repeated until either the year counter reached 200 and the entire stand was harvested, or no longer held any viable trees.

For each series of simulations described below, we replicated the treatment in 10 different even-aged stands, 5 stands for non-thinned controls and 5 stands for PCT. Each stand was originated using a unique set of random number seeds, however, identical random number seeds were employed for pairs of control and treatment stands. As such, each pair of stands generated identical dynamics up until PCT was implemented, confirming that any differences observed were due to treatment effects and not stochasticity.

To make our model relevant to forestry practices, we converted tree size to volume (V) by the following physical equation:


(12) $$V=\frac{S}{D}$$

where: D = wood density, which is species specific, in this case, for pine, is 455 kg/m3 (https://www.thecalculatorsite.com/conversions/weighttovolume.php).

### 2.3 Model implementation

For each simulation, we considered two different management tactics: (i) no pre-commercial thinning (from here on referred to as Control) and (ii) PCT as described in section 2.2.

To demonstrate the utility of our approach we used the simulations in the following manner:

(i) We plotted mean volume for trees from both thinned and control plots to demonstrate that stand-level compensatory growth is determined by enhanced growth of remaining trees;
(ii) We plotted stand volume curves, over time, to show that overcompensation occurs relative to the Control stands;
(iii) We varied the size dependent growth rate shape parameter θ (Equation 4) to evaluate how it impacts the degree of overcompensation;
(iv) We varied (0.2, 0.25, 0.3, 0.35 and 0.4) the inter-tree competition shape parameter (τ’) to evaluate how it impacts growth curves;
(v) We varied the timing of thinning (at years 15, 30, 45 and 60) to evaluate impact on overcompensation;
(vi) We varied the intensity of PCT (25, 50 and 75% removal) to evaluate their impact on overcompensation. The intensity of thinning is defined as the rate (or % of removal) at which the number of trees are removed: thinning intensity (%) = number of trees removed/stand density x 100. When the stand density is fixed in our model experiment, the absolute density can be directly calculated from the thinning intensity;
(vii) To evaluate the impact of thinning protocols we ran a further set of simulations wherein PCT was randomly applied to trees. In both sets of simulations (i.e., smallest quartile removal and random removal) the size of each of the culled trees was included to determine subsequent average tree size and thus impact on growth of remaining trees (Equation 6). However, since the model is aspatial, we did not calculate removal of any specific tree on growth of near neighbors.

### 2.4 Data analysis

Standardized relative growth (SRG) (Equation 13) is a proper indicator of the status of compensatory growth. We calculated RG to show the pattern of stand growth in thinned plots respect to control as follows:


(13) $$SRG\%=\left(\frac{V_{Thinned}-V_{Control}}{V_{Control}}\right)x100$$

Where V_thinned_ and V_Control_ are stand volumes for thinned and control plots, respectively. By definition, under compensation is denoted when SRG is less than 0, exact compensation happens when SRG equals to 0, and overcompensation is showed when SRG is greater than 0.

## 3 Results

i) Does PCT generate compensatory growth at the individual tree level?

**Figure 3:**
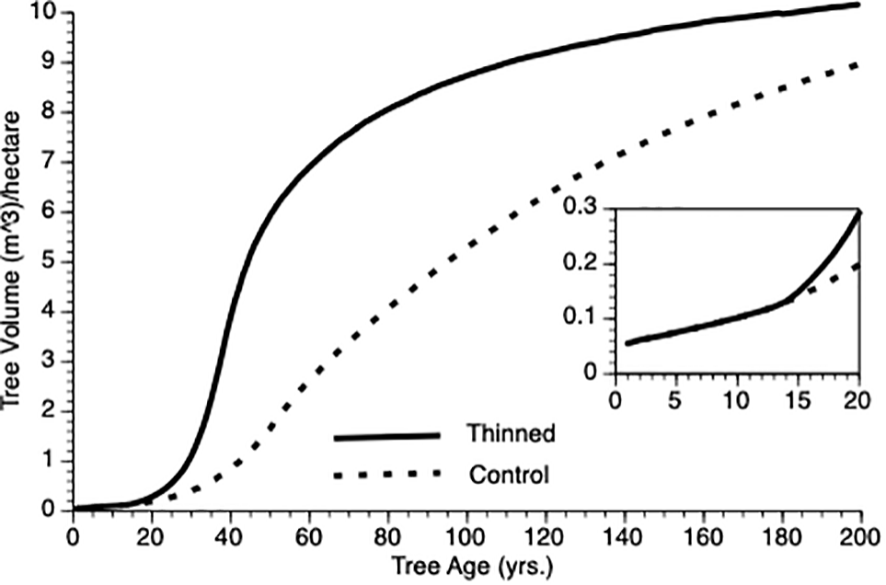
Mean tree volume over time from TAG simulated stands that were either subjected to pre-commercial thinning of 25% at year 15 (solid line) or left unthinned (stippled line).

**Figure 3** shows the change in mean volume of trees over time. In this figure, thinning occurs at Year 15 in the thinned plots (see inset). Several features of this plot should be noted. First, as expected, trees in the thinned and control plots grew at an increasing rate for nearly 50 years. Second, following thinning, mean volume of trees in thinned plots exceeded those from control plot trees for the length of the simulation. Third, the inflection point in the growth curve for trees in the thinned plot sits to the left of that for trees in the control plots (ca. year 40 vs 50). Eventually, however, both plots show trees approaching asymptotic size.

In the Introduction, we posited that compensatory growth could be generated by adaptive life history response to internal and external tree state. Equations 1 and 3 (α and φ, respectively) predict enhanced proportional investment in growth, primarily from harvested resources but also from reserves. Small trees that are released from intense competition due to thinning (natural or otherwise) should invest proportionately more in growth than do controls because increased harvest reduces their risk of reserve depletion. To test this supposition, we employed two metrics mean φ and coefficient of variation for φ (CV), which we evaluated for the 25 years following PCT. As expected, mean φ values for thinned trees exceeded those for controls (0.57 vs 0.44 m^3^, N = 125 i.e., 5 replicates per treatment x 25 years). This confirms that overcompensation is due to thinned trees investing proportionately more of their harvested energy into growth as compensatory growth (Mangel and Munch, 2005) i.e., increased growth performance cannot simply be ascribed to reduced competition among trees. We noted one other feature of φ that was not obvious during theory development. Coefficient of variation was greatly reduced in thinned stands versus controls (0.15 vs 0.55, N = 125) (see Bose et al., 2018). Our interpretation is that thinned trees are unconstrained from consistently investing adaptively in growth whereas being risk adverse, control trees only do so in sufficiently good weather years (see Equation 3). As risk aversion (H) and variability in weather increases, so should the relative difference in φ mean and CV, initially and then they should converge as the perceived risk to thinned individuals impacts φ. However, it is important to recognize that both thinned and control trees do respond adaptively to their circumstances, however, control trees are constrained from expressing consistent investment as discussed here.

Finally, differential volume increases between thinned and control plot trees that we observed is not guaranteed; under some conditions we would expect individual trees to allocate increased resources (via reduced competition in thinned plots) to reserves instead of increased growth thus eliminating differences among trees between plots. This may be especially true if stress is severe or long lasting (e.g., Lv et al., 2022).

ii) Does the TAG model generate stand-level overcompensation?

**Figure 4:**
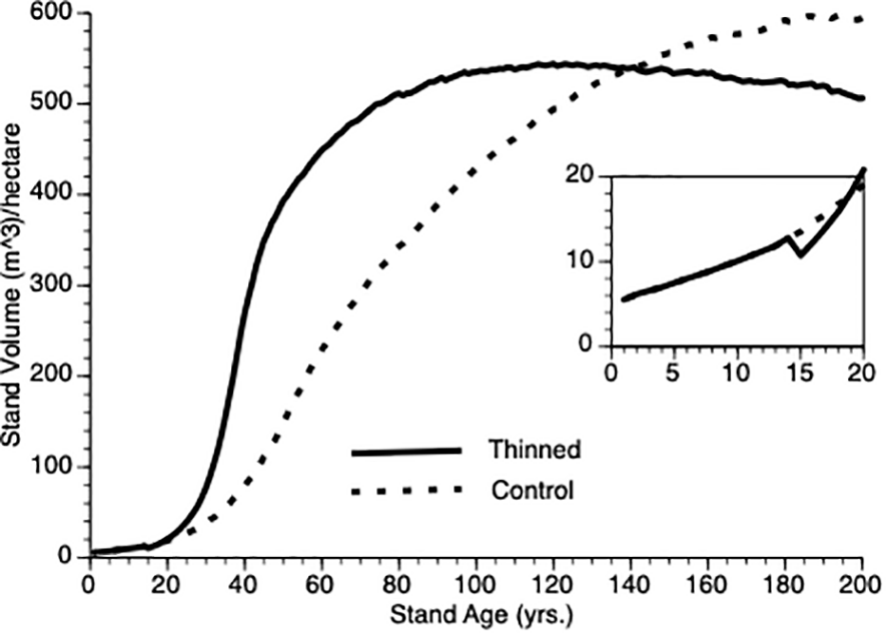
Stand volume over time from TAG simulated stands that were either subjected to pre-commercial thinning of 25% at year 15 (solid line) or left unthinned (stippled line).

**Figure 4** compares stand-level output over time for (non-thinned) control and for stands pre-commercially thinned at 15 years after onset. Despite a 25% reduction in tree population size at that point, the thinned population stand volume exceeds that of the control population within 5 years of thinning, clearly demonstrating overcompensation, faster than has been found empirically (see Discussion for plausible biological explanations for this quantitative discrepancy). Note, the inflection point in the growth curves sits to the left (ca. year 40) for the thinned plot versus the control plot (ca. year 60). Also, note that given sufficient time, the TAG model predicts that control stand volume will meet and exceed that of the thinned stand as the smaller number of trees in the latter express asymptotic growth though we are not aware of any data sets that are followed over sufficiently long periods to confirm this prediction.

**Figure 5:**
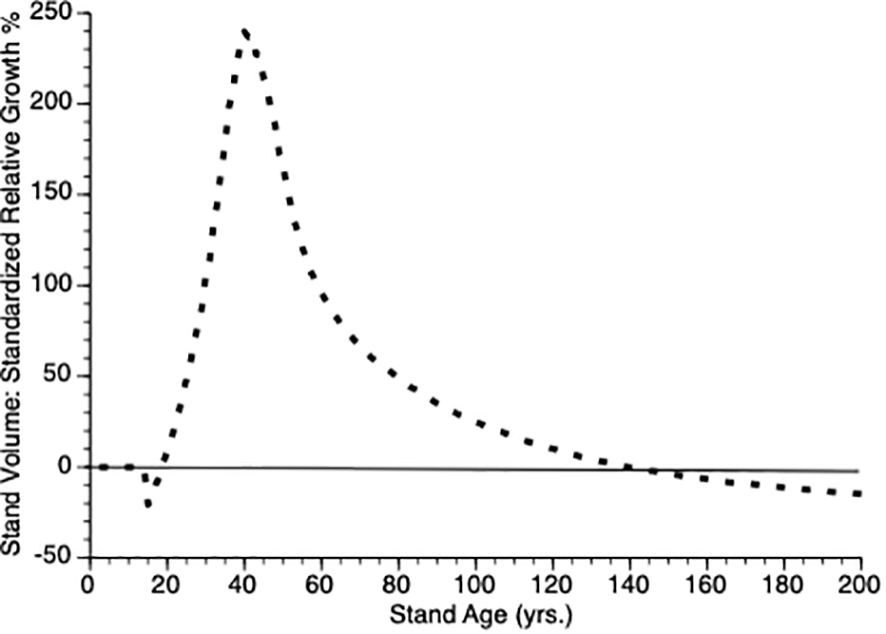
Standardized relative growth rates for stands over time from simulated stands that were either subjected to pre-commercial thinning of 25% at year 15 (stippled line) or left unthinned (solid line).

Another way to visualize the impact of PCT is to plot relative growth for each treatment (Equation 12). This is shown in **Figure 5**. It is obvious that soon after PCT is applied that the thinned stand shows overcompensation, eventually by 300%. Though this value is higher than is found in nature, the qualitative pattern holds i.e., initial performance of thinned plots falls below that controls, which is followed by overcompensation, which peaks at the inflection point in the thinned plot growth curve while the control plot continues to grow at an accelerated rate for another 20 years. Eventually, control plots show higher gross volume simply because they harbor more trees.

iii) How does the size dependent growth rate shape parameter θ impact stand level productivity?

**Figure 6:**
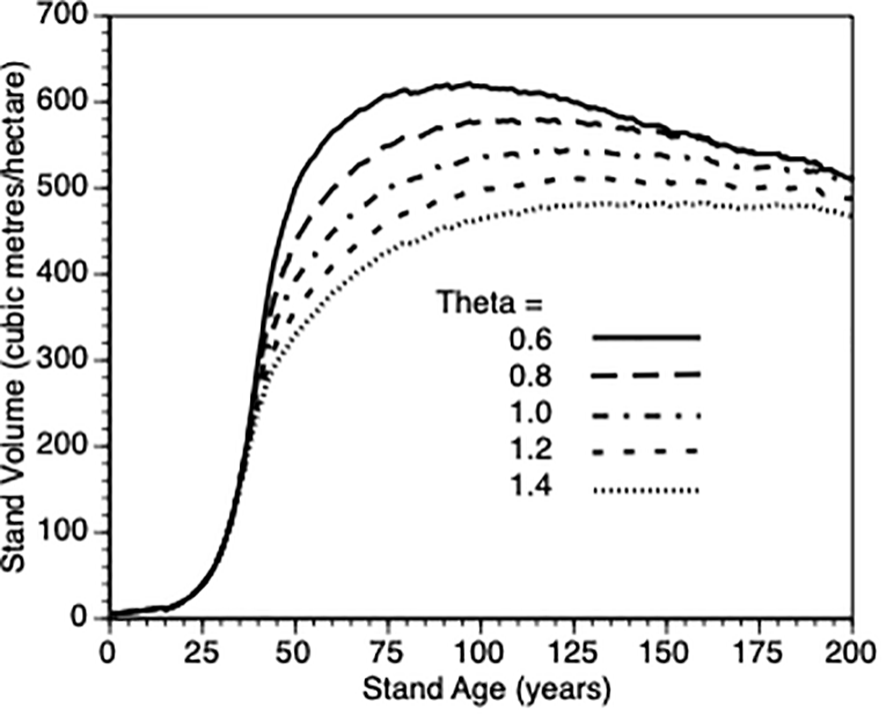
The impact of within-tree size dependent growth (θ = 0.6, 0.8. 1.0, 1.2, and 1.4) on stand volume from TAG simulated stands that were subjected to pre-commercial thinning of 25% at year 15.

We varied θ from 0.6 to 1.4 in steps of 0.2 and plotted stand volume (m^3^)over maximum lifetime of stands (200 years) that were thinned by 25% at Year 15 (**Figure 6**). In response, every stand produced sigmodial growth. This is not surprising since the growth model for individual trees is based upon a logistic growth curve that is modified by the aforementioned life history adjustments and changes in stand density. The best performing sites were those whose trees are relatively insensitive to their own tree size when such trees are small. When such trees are partially released from inter-tree competition via PCT, they gain more benefit than those trees whose growth is suppressed due to within tree competition for resources. It is not possible to directly compare the effect of this parameter for thinned trees against default control trees since the latter trees would also be affected by a change in this shape parameter. To get a sense of this parameter on the PCT effect, we compared stand volume for thinned versus control at Year = 50 for θ 0.6 vs 1.4 in both types of stands. We observed a nearly 200% increase in both cases (200% vs 190%, respectively) i.e., the PCT performance effect is relatively insensitive to the within-tree growth shape parameter though it clearly impacts tree growth (**Figure 6**).

iv) Does inter-tree competition impact stand level productivity?

**Figure 7:**
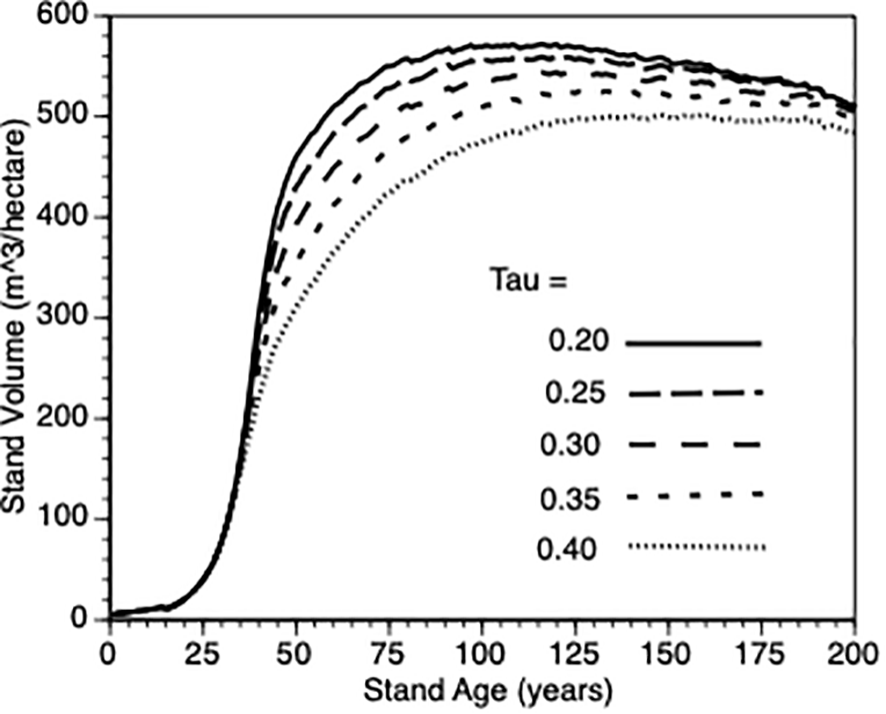
The impact of inter-tree competition when the shape parameter (τ’) varied from (0.2, 0.25., 0.3, 0.35 and 0.4 - see Equation 5) on stand volume from TAG simulated stands that were subjected to pre-commercial thinning of 25%.

To provide more insight into stand growth, the next plot (**Figure 7**) shows stand performance sensitivity to within-stand inter-tree competition when the shape parameter (τ’) varied from 0.2 to 0.4. As expected, stand volume increased more rapidly when inter-tree competition was relaxed. As in 3.iii, it is not possible to compare directly with the default Control stands because their values would also be impacted by τ’. Further, as directly above, to get a sense of this parameter on the PCT effect, we compared stand volume for thinned versus control at Year = 50 for τ’ 0.2 vs 0.4 in both types of stands. In both cases (3.0 vs 2.8., respectively) i.e., the PCT effect is relatively insensitive to the within-stand, inter-tree competition shape parameter.

v) Does the timing of thinning impact overcompensation?

**Figure 8:**
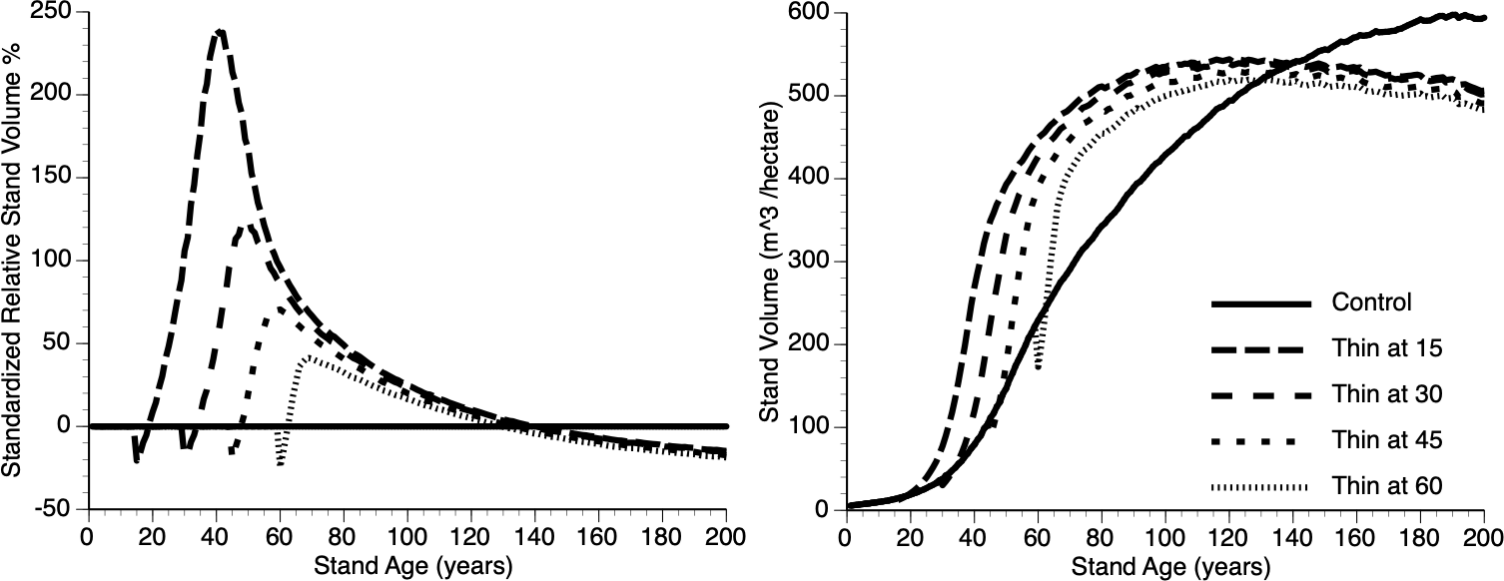
The relative (left) and absolute (right) impact of timing of thinning (Year - 15, 30, 45, 60 and Control) on stand volume from TAG simulated stands that were subjected to pre-commercial thinning of 25%.

**Figures 8**i, ii shows absolute and relative growth curves, respectively for stands at 5 levels of timing (Years 15, 30, 45, 60 and 0 (Control)). Several features can be seen: First, all plots show sigmoidal growth as expected (**Figure 8**i), second, all thinned plots show overcompensation (**Figure 8**ii) and third, plots thinned early, perform better than plots thinned late (e.g., compare year 15 vs year 45 thinned plots) both in absolute and relative terms.

vi) Does the thinning intensity impact overcompensation?

**Figure 9:**
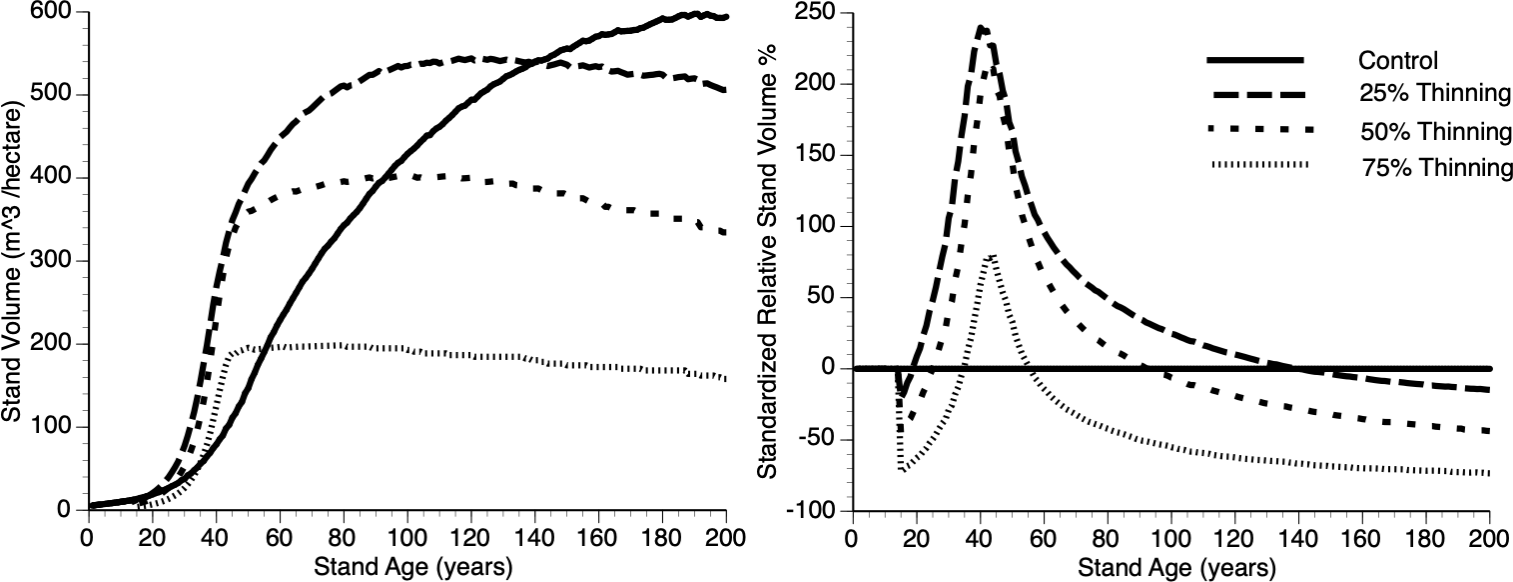
The absolute (left) and relative (right) impact of thinning (25%, 50%, 75% and Control (0%)), at Year = 15, on stand volume from TAG simulated stands that were subjected to pre-commercial thinning.

**Figures 9**i, ii shows absolute and relative stand level performance, respectively as a function of thinning intensity, which varies from 0 to 75% removal of the smallest trees at Year 15. Several features can be gleaned from these figures. First, as percentage culling increases there is an inverse response in terms of asymptotic value (**Figure 9**i). This is simply due to a smaller population of trees resulting from increased thinning, with all trees approaching their maximum size and thus the concomitant impact. Second, immediately following thinning, there is undercompensation, at the stand level, and it takes longer for stands to move into the overcompensation region under heavy thinning intensity (**Figure 9**ii). Finally, under heavy thinning intensity, there is a very short time window where overcompensation occurs (**Figure 9**ii). Again, this is due to the small number of trees in the thinned plots approaching their asymptotic size and, as such, gross volume declines slowly along with the occasional death of one of those trees.

vi) Does thinning protocol impact stand level growth?

**Figure 10:**
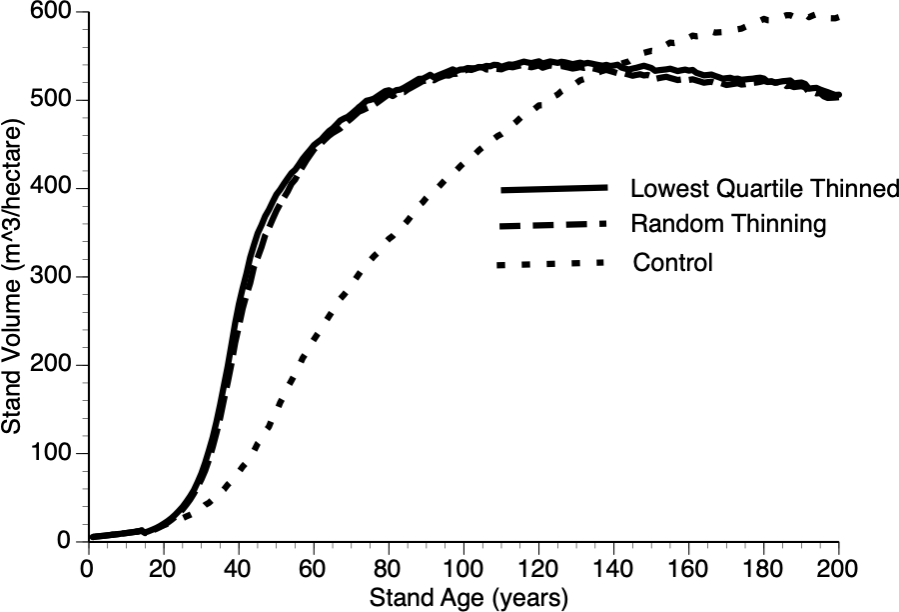
The impact of thinning protocols (smallest quartile, random removal of 25% of living trees and Control) on stand volume from TAG simulated stands that were subjected to pre-commercial thinning at year 15.

**Figure 10** compares growth in thinned and control plots when two different strategies were employed, the default removal of the smallest quartile of trees versus random removal of trees at the year of thinning. The resultant difference from these two approaches is minimal with both tactics outperforming the controls. Our reasoning for this non-obvious, minor-protocol effect is as follows: removing the smallest quartile of trees necessarily increases the mean size of the remaining trees thus reducing the competitive ability of survivors - recall that individual performance depends upon relative size (Equation 6 and section 3.iv).

## 4 Discussion

Compensatory growth is a well-known phenomenon for a wide variety of organisms from both the plant and animal kingdoms (e.g., lima beans (*Phaseolus lunatus*) - Bustos-Segura et al., 2022 and cattle (*Bos taurus*) - Keady et al., 2021). This includes trees both at the individual and stand level (e.g., Li et al., 2018). However, as has been noted on several occasions, the dynamics associated with compensatory growth of forests can vary dramatically depending upon intrinsic (i.e., species specific) aspects as well as site characteristics. Thus, there is a need to provide general models that can explain this phenomenon and can be used to optimize forest growth potential. In this paper, we developed a simple model of tree growth, TAG, that is based upon life history theory for long-lived trees and can be extrapolated to the forest level. Moreover, the TAG model can incorporate PCT to take advantage changes in growth in response stand density.

### 4.1 The model

The primary goals of this paper are: (i) as a proof of concept, develop a biologically-based theoretical model for tree and stand growth (TAG), (ii) demonstrate that the TAG model generates stand dynamics similar to that found in nature, (iii) demonstrate that stands that experience thinning can overcompensate under some conditions and (iv) explore the sensitivity of the compensatory growth to some easy-to-implement management tactics (e.g. intensity of thinning) as well as inherent characteristics of different tree species (e.g. sensitivity to crowding).

Based upon evaluation of the TAG model, the following insights emerged: (i) compensatory growth, following thinning (natural or otherwise) occurs readily especially early on in the life of a stand, (ii) the window for overcompensation narrows inversely with the intensity of PCT simply because increased performance by individuals is negated by reduced numbers of such, (iii) early PCT is beneficial because increased investment in growth is most readily observed during the accelerating part of the growth curve, (iv) results from the various sensitivity analyses were in line with expectations and (v) relative performance of thinned stands were higher than those observed in nature (see below). Finally, an important insight from employing individual trees was that the ‘catching up’ response of surviving trees, post-PCT (**Figure 2**), can be explained by such individuals gaining greater access to limiting resources and investing more of those resources into growth because it is safe to do so i.e., low risk of draining reserves. We would not have identified this crucial mechanism had we not employed an agent-based, life-history driven approach.

As shown in section 3ii, the overcompensation generated by the TAG model was nearly two to three times larger and two to five times faster, depending upon PCT treatments and site conditions, compared to observations from the Shawnigan Lake trial (Li et al., 2018), as has been observed in managed stands (**Figure 4**). There are several biologically plausible explanations for this discrepancy. First, values from the simple inverse relationship between crowding and growth (Equation 5) that we employed may have been too high. It is possible that other factors in the environment (Bennett et al., 2017) may ameliorate stress from crowding. Second, we employed an aspatial model, for heuristic reasons, but in doing so, we implicitly assumed a sized-biased, scramble competition for those resources that are forfeited by the now-culled trees (Kato et al., 2007). This might be a reasonable approximation for mobile organisms but it is too simplistic for sessile ones and would lead to overestimates of resource accrual within the stand (Husband and Barrett, 1996). Third, we assumed no limiting resources (e.g. nitrogen) to growth other than those competed for (e.g. energy, water, etc.) in the aforementioned competition. Fourth, as is often the case in heuristic models, we assumed near instantaneous access to resources that were freed up via the thinning process. Fifth, while there is an implicit effect of year-after-year stress in our model via Equations 1-3, there is no explicit assumption that long-term stress has cumulative effects, which would make compensatory response more difficult (e.g., Lv et al. 22). If any or all of the above assumptions were relaxed, that would lead to slower and less uptake of resources by trees in the thinned plots and would necessarily reduce resource harvest and the LTH allocation value for the parameter φ, which, in turn, would reduce magnitude and speed of overcompensation. Further, we assumed that survivorship would be equal in both thinned and control plots however, windthrow could increase soon after thinning, further reducing the number of trees and thus initial thinned stand productivity (Bose et al., 2018). Finally, again, for heuristic reasons, we employed simple baseline growth curves whereas further modification using specific allometric descriptors (e.g., Kramer et al., 2018) may have reduced the gap even further between thinned and control stands though we would still expect to observe the signature of overcompensation. All of these simplifications could be remedied without changing the tenor of our approach but that would reduce the generality of our model, which was the primary goal of this proof-of-concept exercise.

Further to the discussion above, it is possible to increase the reality of the TAG model without losing its generality however doing so would sacrifice some of its elegance. For example, suppose we wish to move from single-species to mixed-species stands; that would require that we rewrite Equation 5 from the original $\tau =1-(\frac{N_{y}}{N_{0}}{)}^{\tau '}\psi$ (Equation 5) to $\tau =1-(\frac{\sum_{y=1}^{V}v_{y}}{\sum_{y=1}^{V}v_{0}}{)}^{\tau '}\psi$ (equation 5.1) and Equation 6 from the original $\psi_{s,n,y}=\frac{s_{n,y}}{{\bar{s}}_{y}}$ (Equation 6) to $\psi_{s,v,n,y}=\frac{s_{v,n,y}}{\sum_{v=1}^{V}{\bar{s}}_{v,y}\lambda_{v}\frac{v}{N_{y}}}$ (equation 6.1).

The interpretation is that we now have accounted for those different species (or varieties) of trees of which there are V types (v = 1, 2, 3…V) each with average size s in a given year y which must be further weighted by both their interspecific competitive ability (λ_v)_ against the focal individual and their relative density. To account for different size classes would require another summation term that would cycle through those size classes within each tree variety.

As can be seen from this discussion, it is certainly possible to modify the TAG model to include diverse forests, however the complexity from doing so increases dramatically particularly with regard to statistical analysis and/or visualization given that we would have now added more dimensions to the problem. The same can be said for some of the other simplifications that we have employed, for example, the assumption of a non-spatial world. Even if spatial representation is included, there are practical limits to degree of resolution. For example, in Guignabert et al. (2023) spatially-explicit, forest dynamics model, seedlings were binned into size classes within 10m^2^ plots to reduce computational challenge.

At least two possible criticism of the TAG model need to be reconciled. First, TAG is generic and cannot be directly employed to make silvicultural decisions, for example, the exact amount of thinning that should be applied to a particular stand. We fully agree that this model is only the first step toward developing a comprehensive PCT silviculture system and as such we have described it as proof of concept. At this point, we cannot say how broadly applicable TAG will be across a range of tree species an environments however most of the parameters that we employed are measurable thus it should yield testable predictions. Second, we have made a number of reasonable assumptions while deriving the TAG model some of which are untested. We hope that development of this novel theory will generate interest in parameters that until-now have garnered little attention.

On the other hand, the major contribution of the TAG model development is to confirm that a simple theoretical model can provide meaningful explanation and predict the compensatory growth phenomenon including overcompensation in post-thinning forest stands and the possibility of stand productivity enhancement. In particular, overcompensation has been difficult to understand and explain in the past but here it is seen as the simple outcome from enhanced resource access and disproportionate investment resource accrual into growth by trees in thinned plots. As we have noted, the simulation results are consistent with existing observations in both short and long term, without digging dept into very detailed physiological processes or waiting for corresponding empirical observations become available. The results presented in this paper reinforce the conclusions from the statistical approach of the TreeCG model with a verification from running the operational TASS model (Li et al., 2022b). These duel approaches can help speed up our understanding and predictability of complex forest dynamics in both natural and managed stands.

### 4.2 State dependence is key

A feature demonstrated in the TAG model development is the modeling approach of state-dependence (e.g., Li et al., 2020), which means that tree growth is determined by yearly changes in internal and external/environmental conditions/states, such as the internal states of tree size, age, and energy reserve, and the external states of available level of nutrients and fluctuations in weather variables. The advantage of this state-dependent modeling approach is the possible incorporation of adaptive growth/responses of trees during their long lifespan, with which the growth of trees is no longer a fixed function of tree age and site index as shown in many forest growth and yield models such as VDYP7 of BC and GYPSY of AB, Canada, but rather the results of a continuous trade-off process within a changing environment as indicated by the life-history theory. In this case, plant growth is closely related to resource capture and allocation over time (Thomas, 2002). In this paper, we finessed (i.e., no explicit optimization) state-dependent dynamic life history responses (Clark and Mangel, 2000) via the α, β and φ functions to describe the adaptive responses of individual trees to changing environment and this approach generates understandable, stand-level growth patterns. However, even using this finessed approach requires detailed calculations that take more time than that from conventional growth and yield models, but each simulation run can still be completed within a few seconds using a personal computer. The incorporation of these adaptive growth functions makes it suitable for exploring the effect of other time sensitive management operations such as fertilization and pruning, as well as addressing climate change related issues. (Note that our approach makes assumptions regarding the shape and direction of tree responses to their states. An assumption-free approach to calculating optimal response requires searching the fitness surface via backwards induction as explained by Mangel, Clark and others (Mangel and Clark 1986; McNamara and Houston, 1986). Another option for adding more realism and searching resultant complex fitness surfaces would be to employ genetic algorithms (e.g. Chubaty et al., 2013) again with the possible expense of losing some heuristics.

The state-dependent modeling approach is relatively new and unfamiliar to many foresters but promising in a sense of not only harmonizing the feature of conventional growth and yield modeling approach, but also opening the door for investigating how trees might respond to their changing environments, so as to be capable of addressing additional related issues. The state-dependent modelling approach has been widely employed in the field of behavioral ecology for representing how the behavior of animals could be explained as a result from response to specific environmental conditions. Many examples can be found from elucidating response to danger in tiny parasitoid wasps (*Asobara tabida*) (Roitberg et al., 2009) to explaining flexible torpor in insectivorous bats (Fjellddall et al., 2023) to evaluating impact of anthropogenic disturbance on adaptive foraging in beluga whales (*Delphinapterus leucas*) (McHuron et al., 2023). The research approach from behavioral ecology has recently been expanded to plants (Cahill, 2015; Simon et al., 2016), and this multi-disciplinary approach has demonstrated the benefits in enhancing our understanding of the dynamics of various ecosystems. Our current study is an example of this approach.

### 4.3 Future applications

The general stand growth patterns simulated by our TAG model can be widely applied as long as the growth curves from natural stands display a sigmoid shape. Thus, the calibration of this life history theory-driven TAG model could be much simplified compared to statistics-driven models such as TreeCG and hence widely applied to many jurisdictions without detailed stand growth relationships. This feature could serve as a complement to experimental approaches in a sense of providing a theoretical prediction even before implementing these experiments. As such, simulation results from carefully designed TAG model experiment could help address silviculture-effect related issues such as optimal thinning strategy leading to maximized productivity and optimal spacing in achieving the goals of stand density management, and could provide useful insights for improved field experiment designs, as well as for supporting forestry policy development and practical forest management decisions to mitigate the challenges from wood supply shortage. This modeling approach can also be applied to investigating the effect of other disturbances such as fire and insect pests, as well as other species of plants and animals with sigmoid growth pattern, especially those with long lifespans.

As an example of future applications of the TAG model, consider climate change. As a starting point, we envision the following in an attempt to retain generality while focusing on specific problems: in our ‘weather’ parameter, we assumed that climate is stable and weather can be drawn from a likewise stable distribution. To deal with climate change, we would draw from two two-dimensional matrices (ς_s,y_ and ς_f,y_) based upon data from Coupled Model Intercomparison Project (CMIP6) (https://cds.climate.copernicus.eu/cdsapp#!/search?type=dataset&text=CMIP6) or similar, that assumes a changing climate rather than a stable one. The challenge in doing so will be to extrapolate change in tree growth performance as a function of changing climate.

Finally, as is the case with many theoretical studies, we have generated many new questions, especially those related to PCT and compensatory growth. A few of those include: (i) Since the adaptive compensatory growth response depends greatly up release from crowding stress, can this feature be generalized (see Equations 5 and 6) or will they be unique among species and sites? (ii) Similarly, will such stress show as simple additive functions or will they display emergent properties in mixed stands? (iii) Since climate will change necessarily advance much faster than the evolution of tree life history traits unlike short-lived plants (e.g. Acoca-Pidelle et al., 2023), can we employ models such as TAG that include contemporary tree life history values to navigate the many management obstacles that await us, at least in the near term? (iv) Can the post-thinning stand dynamics be extended to post-disturbance, including fire and post-pest-driven stand dynamics, especially if trees are incapable of distinguishing the causes of partial mortality from different types of disturbances? (Note that Arimura (2021) reviewed plants’ response to herbivore elicitors that triggered plants unique defense mechanism that was identified as one of the compensatory growth mechanisms summarized by Prins and Verkaar (1992)). These are just a few of the challenges to sustainable forest management, however, the adaptive nature of tree growth presents an opportunity to develop effective dynamic strategies.

## 5 Conclusions

By including state dependence in an agent-based adaptive growth model (TAG), it was possible to demonstrate overcompensation by thinned stands in computer simulations. This is largely driven by thinned trees investing proportionately more of their increased resource allocation (due to reduced competition following thinning) because it adaptive to so. These results have implications for sustainable forest management.

## Data availability statement

The raw data supporting the conclusions of this article will be made available by the authors, without undue reservation.

## Author contributions

BR: Conceptualization, Formal analysis, Investigation, Methodology, Software, Visualization, Writing – original draft, Writing – review & editing. CL: Conceptualization, Data curation, Formal analysis, Investigation, Methodology, Project administration, Resources, Visualization, Writing – original draft, Writing – review & editing. RL: Conceptualization, Data curation, Formal analysis, Investigation, Methodology, Visualization, Writing – original draft, Writing – review & editing.
